# *Brettanomyces bruxellensis* Strains Display Variable Resistance to Cycloheximide: Consequences on the Monitoring of Wine

**DOI:** 10.3390/microorganisms13112597

**Published:** 2025-11-14

**Authors:** Laura Olazabal, Quentin Dapzol, Warren Albertin, Cécile Miot-Sertier, Magali Deleris-Bou, Anita Boisramé, Marguerite Dols-Lafargue

**Affiliations:** 1 Univ. Bordeaux, INRAE, Bordeaux INP, Bordeaux Sciences Agro, OENO, UMR1366, ISVV, F-33140 Villenave d’Ornon, France; laura.olazabal@inrae.fr (L.O.); warren.albertin@u-bordeaux.fr (W.A.); cecile.miot-sertier@u-bordeaux.fr (C.M.-S.); 2 SPO, INRAE, Institut Agro, Univ. Montpellier, 34060 Montpellier, France; anita.boisrame@agroparistech.fr; 3 Lallemand SAS, 31700 Blagnac, France; mbou@lallemand.com; 4 AgroParisTech, Université Paris-Saclay, 91120 Palaiseau, France

**Keywords:** *Brettanomyces bruxellensis*, wine, spoilage, selective medium, cycloheximide

## Abstract

*Brettanomyces bruxellensis* is a yeast that causes spoilage in red wines due to its ability to produce volatile phenols, compounds associated with major sensory defects. Specific monitoring of low populations of this species in complex ecosystems such as wine during fermentation or aging often relies on plating onto selective media supplemented with cycloheximide. However, the variability of *B. bruxellensis* sensitivity to this antibiotic needs to be better characterized. A collection of 175 *B. bruxellensis* strains was, thus, grown on YPD medium supplemented with increasing concentrations of cycloheximide (0 to 0.5 g.L^−1^), and yeast development was monitored for 20 days by image analysis. This study revealed significant inter-strain variability, with some strains showing very late or even no growth at high cycloheximide concentrations. The cycloheximide inhibitory effect was also dose- and population-dependent. In addition, colony size was frequently reduced at high doses. Additional tests were conducted on a subset of strains grown in wines with either low pH or high alcohol content or containing sulfur dioxide and then plated in the presence of increasing concentrations of cycloheximide. This revealed a cumulative effect of wine and cycloheximide stresses that resulted in an even higher delay in yeast detection. The results confirm the huge phenotypic diversity of the species and highlight the need to adapt the plates’ incubation time, particularly when the selectivity and the doses of cycloheximide needed are high (samples taken in pre-fermentation phases or during fermentation) or in case of stressful wine analysis, to minimize the risk of false negatives.

## 1. Introduction

*B. bruxellensis* is considered one of the main spoilage yeasts in red wines. It is responsible for the production of volatile phenols, undesirable compounds that can negatively affect the sensory and organoleptic qualities of the wine [1]. Indeed, *B. bruxellensis* has the ability to convert free hydroxycinnamic acids naturally present in wine, mainly p-coumaric and ferulic acids, into 4-ethylphenol (4EP) and 4-ethylguaiacol (4EG) [1,2]. At low concentrations, these compounds have little to no impact on wine aroma. However, above 400 μg.L^−1^, or sometimes less depending on the sensitivity of the tasters [3], they impart unpleasant notes described as leather, horse sweat, or burnt plastic [1,4,5]. This so-called “phenolic character” not only leads to wine rejection by consumers but can also downgrade for a long time the reputation of the winery.

*B. bruxellensis* exhibits substantial intraspecific genetic diversity. In 2018, a microsatellite analysis of 1488 isolates from various geographical origins and ecological niches defined six genetic groups within the species [6]. Subsequent genome sequencing studies confirmed and refined this population structure [7,8,9]. More recently, a combined genomic and phenotypic analysis of 1060 isolates redefined this organization into seven genetic groups [10]. Several studies highlighted associations between genetic groups and the resistance to abiotic stresses encountered in wine, such as sulfites [11,12], ethanol, or acidity [13,14]. Beyond these stress responses, strains also display diverse growth behaviors in wines [15,16].

No efficient curative method exists to eliminate volatile phenols, and preventing wine spoilage, therefore, relies on the ability to detect *B. bruxellensis* early and reliably. Various approaches have been developed to enumerate this yeast. Among culture-independent methods, quantitative PCR assays targeting specific DNA or RNA fragments have been widely explored for the identification and quantification of *B. bruxellensis* [17,18,19,20,21]. For instance, ref. [18] designed a qPCR assay targeting the *rad4* gene, enabling quantification from 31 CFU. mL^−1^. However, they reported substantial variability and the need for repeated measurements. Later studies revealed quantification biases. Ref. [20] showed that qPCR kits could either underestimate or overestimate actual *B. bruxellensis* populations. Indeed, DNA-based qPCR quantifies both live and dead cells, potentially overestimating viable populations [20]. The use of DNA intercalants, such as ethidium monoazide (EMA), enables the quantification of only viable cells [22]. The reported detection limits for qPCR range between 10 and 10^3^ cells. mL^−1^, depending on the assays. Flow cytometry has also been evaluated for *B. bruxellensis* enumeration. Ref. [23] developed a method combining flow cytometry with fluorescence in situ hybridization (FISH). More recently, ref. [24] proposed a multicolor flow cytometry approach applicable to complex samples and combining a metabolic activity indicator (FDA) and a specific anti-*Brettanomyces* antibody. Other methods such as CYTO-3D (COFRAC accredited, no. 1-0207) are also available. These methods can detect populations as low as 10^2^ cells. mL^−1^ within 48 h and provide information on cell viability. They also enable the detection of viable but non-culturable (VBNC) cells, a physiological state reported for *B. bruxellensis* upon exposure to sulfites in wine [19,25,26,27,28,29].

However, while these culture-independent methods offer rapidity, sensitivity, and specificity, they remain costly and require specialized equipment and expertise, limiting their routine application. As a result, despite recent advances in molecular and cytometric tools, culture-based approaches are still widely used in laboratories and wineries. They are simple and inexpensive and enable the detection of extremely low contamination levels, down to 1 colony-forming unit (CFU) per mL of plated wine. Their main limitation is the time required for results, because *B. bruxellensis* typically takes 3 to 6 days to form visible colonies. Most selective media designed for *B. bruxellensis* detection rely on cycloheximide as the selective agent [1,30,31,32,33], while some others rely on lysine as the sole nitrogen source [31] or alternative carbon sources such as maltose, trehalose, or ethanol [30]. Cycloheximide (also called actidione) inhibits protein biosynthesis in many eukaryotes, including *Saccharomyces cerevisiae* [34]. It binds the 60S ribosomal subunit, blocking the initiation and translocation steps of protein synthesis [35]. However, several wine-associated yeasts other than *B. bruxellensis* are slightly resistant to cycloheximide, which can compromise the selectivity of such media. *Hanseniaspora uvarum*, *Hanseniaspora guillermondi*, *Pichia guilliermondii*, and *Schizosaccharomyces pombe* have been reported to grow at concentrations between 10 and 50 mg.L^−1^ [32,33,36]. Ref. [32] report that *H. uvarum* and *H. guillermondi* tolerated cycloheximide doses even up to 100 mg.L^−1^. Other species, including *Torulaspora delbrueckii*, *Candida pararugosa*, *Zygosaccharomyces bisporus,* and *Zygosaccharomyces rouxii*, may also grow at low concentrations (≤10 mg.L^−1^) [32]. Thus, resistance to cycloheximide varies depending on the species, strains, dose, and culture medium, making selectivity relative [33,36]. In practice, “standard” concentrations of 50–100 mg.L^−1^ do not guarantee the exclusive detection of *B. bruxellensis*. As a result, to improve selectivity, some laboratories use cycloheximide concentrations up to 0.5 g.L^−1^ to inhibit competitors. However, ref. [30] showed that some *B. bruxellensis* strains were inhibited above 10 mg.L^−1^. In MMV medium (mineral maltose supplemented with 50 mg.L^−1^ cycloheximide and 500 mg.L^−1^ sorbic acid), only one of five tested strains grew after 12 days of incubation. Conversely, refs. [32,37] reported *B. bruxellensis* growth at 100 mg.L^−1^, although slower than in control conditions without cycloheximide. Ref. [32] further showed that a higher cycloheximide dose could reduce colony size in some strains, while others were unaffected. After 16 days of incubation, colony size was comparable between the control and cycloheximide-supplemented plates, even at 100 mg.L^−1^. Despite the limited number of strains in each study, these results suggest considerable *B. bruxellensis* phenotypic diversity regarding cycloheximide tolerance.

The objective of this study was, therefore, to perform a large-scale phenotyping of *B. bruxellensis* sensitivity to cycloheximide. We examined the behavior of 175 strains in YPD medium and complemented this screening with wine-grown yeast assays on a subset of representative strains. A concentration range from 0 to 0.5 g.L^−1^ was tested, the highest dose corresponding to that commonly used in some laboratories to monitor *B. bruxellensis* in wine samples collected during early winemaking stages.

## 2. Materials and Methods

### 2.1. Yeast Strains

A total of 175 *B. bruxellensis* strains representative of the genetic diversity of the species [6,9,10] were included in this study. The isolates originated from diverse geographical origins and ecological niches, including wine, beer, cider, kombucha, and tequila. Strains were obtained from the following collections: the CRBO collection (Microbiological Resources Center Oenology, Bordeaux, France), the CBS collection (Fungal Biodiversity Center, Utrecht, The Netherlands), the YJS collection (Laboratory for Molecular Genetics, Genomics and Microbiology, Strasbourg, France), the ISVV collection (Institute of Vine and Wine Sciences, Bordeaux, France), the URV collection (Rovira i Virgili University, Spain), the ISA collection (Instituto Superior de Afronomia, Lisboa, Portugal), the PYCC collection (Portuguese Yeast Culture Collection, Lisboa, Portugal), the UNINA collection (University of Naples Federico II, Naples, Italy), the IFV collection (French Institute of Vine and Wine, Val de Loire regional center, France), the WLP collection (White Labs, USA), the NCAIM collection (National Collection of Agricultural and Industrial Microorganisms, Budapest, Hungary), the UCD collection (UC Davis Culture Collection, Davis, USA), the Foggia collection (University of Foggia, Italy), the MUCL collection (BCCM/MUCL Agro-food and Environmental Fungal Collection, Louvain-la-Neuve, Belgium), the NCYC collection (National Collection of Yeast Cultures, Norwich, UK), the UWOPS collection (Culture Collection of the University of Western Ontario, Canada), the DBVPG collection (Industrial Yeasts Collection, Perugia, Italy), and the InterRhone collection (Avignon, France). Details of all strains used, including strain codes and their genetic group assignment, are provided in Supplementary Materials Table S1. For assays in wine, a subset of 10 representative strains covering the main genetic groups was used (L0611, CBS-2499, L0422, L0417, L14190, L0424, L1710, L1733, L14165, and L1754).

### 2.2. Cycloheximide Sensitivity Assays

**Figure 1:**
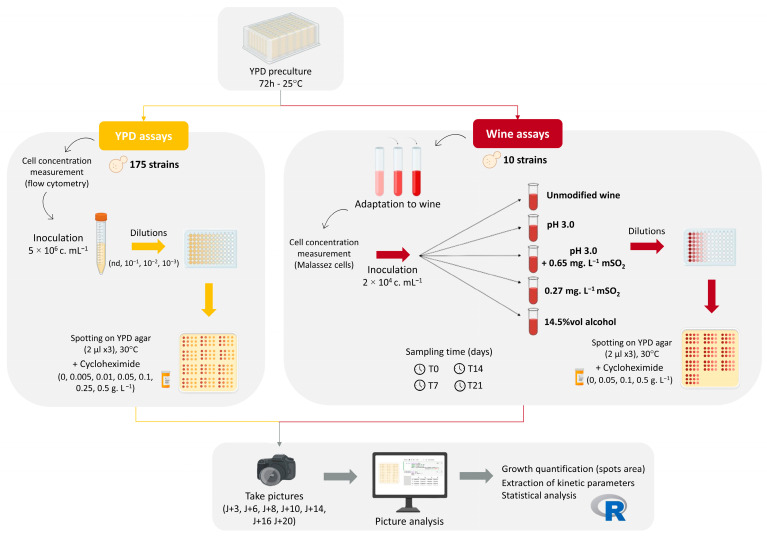
Experimental workflow for cycloheximide sensitivity assays in *B. bruxellensis*. For YPD assays (left, yellow panel), 175 strains were first grown in liquid YPD medium before spotting on YPD agar supplemented with seven cycloheximide concentrations. For wine assays (right, red panel), 10 representative strains were cultivated in a Cabernet Sauvignon/Merlot base wine under five oenological modalities then spotted on YPD agar with four cycloheximide concentrations. In both assays, growth was monitored by imaging, and spot areas were quantified to extract phenotypic parameters.

The assays performed are summarized in Figure 1.

#### 2.2.1. Assays in YPD Medium

After thawing, the strains were reactivated by plating on solid YPD medium (yeast extract 10 g.L^−1^, peptone 10 g.L^−1^, glucose 20 g.L^−1^, agar 20 g.L^−1^). Each strain was then grown for 72 h in liquid YPD medium at 25 °C. Cell concentrations were determined by flow cytometry (CytoFLEX, Beckman Coulter, Fullerton, CA, USA) and adjusted to 5 × 10^6^ cells. mL^−1^ in YPD liquid medium. Serial dilutions were prepared, providing four inoculation densities (corresponding to ≈5000, 500, 50, and 5 colonies per drop). Drops were deposited onto YPD agar plates supplemented with different cycloheximide (SERVA Electrophoresis GmbH, Heidelberg, Germany, CAS 66-81-9) concentrations (0, 0.005, 0.01, 0.05, 0.1, 0.25, and 0.5 g.L^−1^).

#### 2.2.2. Assays in Wine

Strains were gradually adapted to the base wine matrix (50/50 blend of Cabernet Sauvignon/Merlot, 12.91% vol alcohol, pH 3.76, no free SO_2_) through stepwise increases of wine proportion (25%, 50%, and 100%) according to the procedure described by ref. [16]. Adaptation tubes were incubated at 25 °C. At the end of adaptation, cell concentrations were estimated by counting onto Malassez cells, and the assays wines were inoculated at an initial population of 2 × 10^4^ cells. mL^−1^. Five experimental conditions simulating oenological stresses were prepared: (i) unmodified wine (original wine), (ii) acidified wine (pH 3.0), (iii) acidified wine supplemented with 7 mg.L^−1^ free SO_2_ (0.65 mg.L^−1^ molecular SO_2_), (iv) original wine supplemented with 15 mg.L^−1^ free SO_2_ (0.27 mg.L^−1^ molecular SO_2_), and (v) original wine enriched to 14.5% vol alcohol. All sulfite concentrations reported in this study are expressed as molecular SO_2_ (mSO_2_) equivalents, which represent the biologically active fraction of free SO_2_. These values were calculated from the corresponding free SO_2_, pH, temperature (20 °C), and alcoholic strength (12.91% vol). To ensure sterility, wines were pasteurized (25 min, 80 °C) before inoculation. The inoculated wines were then incubated at 20 °C throughout the monitoring period. Cultures were sampled at four stressful time points (T0, T7, T14, and T21 days). At each sampling time, serial dilutions were spotted onto YPD plates supplemented with four cycloheximide concentrations (0, 0.05, 0.1, and 0.5 g.L^−1^). In parallel, population levels were also determined by plating onto solid YPD medium without cycloheximide in order to quantify cultivable cells (CFU. mL^−1^) over time in the different wine conditions.

### 2.3. Spotting, Incubation, and Picture Acquisition

For both YPD and wine assays, 2 µL of each dilution was spotted in triplicate onto YPD agar plates (12 × 12 cm) containing cycloheximide or not. Plates were incubated at 30 °C and photographed at regular intervals (every 2 or 3 days for 20 days).

### 2.4. Picture Processing and Data Analysis

Plate pictures were imported into R using the OpenImageR package Version 1.3.0 (https://cran.r-project.org/web/packages/OpenImageR/index.html, accessed on 9 November 2025) together with other specialized libraries (magick, grid, mixtools, MASS, MESS, plyr). A total of 201 plates and 1311 experimental pictures were processed. The analytical pipeline, developed and published by refs. [38,39], consisted of three main steps: (i) a template of drop positions was defined for each plate from the reference picture (last incubation time point, 20 days) by manually selecting the coordinates of each drop; (ii) all intermediate pictures were realigned to this template through a geometric transformation anchored to the four corners of the plate; and (iii) colony growth was quantified automatically by extracting, for each drop, the growth area (in pixels) after background subtraction. These measurements were then used to generate growth kinetics and to extract different parameters:

- The lag phase (days): Defined as the delay before the detection of colonies or spots on plates (corresponding to the time required to reach a growth area > 500 pixels for high inoculum densities and >250 pixels for the lowest inoculum density).
- The maximum growth area (Amax, pixels): Defined as the maximum spot size reached at the end of incubation, reflecting the intensity of growth once colonies are established.

For the YPD screening, lag phases were then normalized by subtracting the lag value obtained with the same strain in the control condition without cycloheximide (0 g.L^−1^). For the wine assays, lag phases were normalized by subtracting the lag value obtained with the same strain sampled after the same residence time (T0, T7, T14, or T21) in the unmodified wine (original wine) and plated on YPD without cycloheximide.

All statistical analyses were performed with R software version 4.3.3 (2024-02-09). Non-parametric Kruskal–Wallis tests followed by Dunn’s post hoc comparisons (from agricolae package) were used to assess differences between groups. In addition, a multifactorial ANOVA including main effects and interactions was conducted.

## 3. Results

### 3.1. Effects of Cycloheximide on B. bruxellensis Growth on YPD Solid Medium

#### 3.1.1. Influence of the Inoculum Density

To evaluate the influence of the initial cell density on the apparent tolerance to cycloheximide, several dilutions of each strain were spotted onto plates containing increasing concentrations of cycloheximide (0 to 0.5 g.L^−1^) using three levels of density per drop: low (≈5–10 colonies/drop), medium (≈50–100 colonies/drop), and high (≈500–1000 colonies/drop). The “undiluted” condition was excluded, as it rapidly saturated the signal and prevented reliable quantification by image analysis.

**Figure 2:**
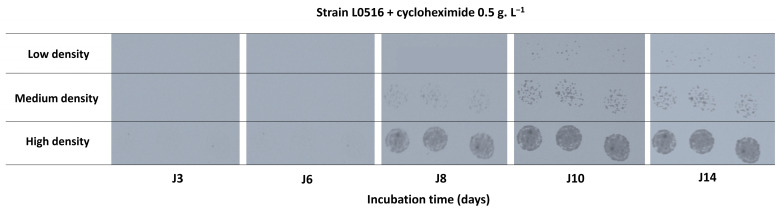
Effect of inoculum density on the growth dynamics of strain L0516 and temporal evolution (J3, J6, J8, J10, and J14) of spots observed in the presence of 0.5 g.L^−1^ cycloheximide at three inoculum densities: low (≈5–10 colonies/drop), medium (≈50–100 colonies/drop), and high (≈500–1000 colonies/drop). The strain L0516 belongs to the genetic group A1.

We observed that the initial inoculum density influenced the delay before growth detection (Figure 2). For strain L0516, in the presence of 0.5 g.L^−1^ cycloheximide, colonies became visible after 8 days (J8) at high densities, whereas at lower density, they were only detected on day 10 (J10). Furthermore, dense inoculum spots quickly saturated the picture, preventing robust surface evolution quantification by image analysis.

Growth recovery on solid YPD medium was also highly variable between strains. In the absence of cycloheximide, the raw lag phase ranged from 3 to 8 days within the collection studied (this point will be further examined later). To better visualize the delay specifically induced by cycloheximide, independently of the intrinsic heterogeneity of baseline lag phases, we analyzed normalized lag phases (Supplementary Materials Figure S1).

At the whole-collection scale, an increase in inoculum density clearly reduced the growth delay induced by cycloheximide. This trend was observed for all cycloheximide concentrations tested, although less pronounced at the lowest doses (Supplementary Materials Figure S1). To avoid visual saturation and simplify results interpretation, all subsequent comparative analyses were performed using the lowest inoculum density (≈5–10 colonies/drop).

#### 3.1.2. Inter-Strain Variability

**Figure 3:**
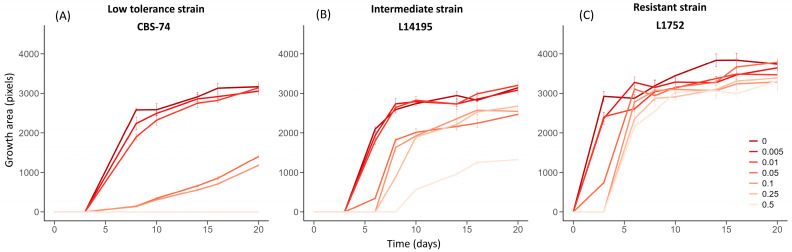
Area growth kinetics for three strains exposed to increasing concentrations of cycloheximide (0 to 0.5 g.L^−1^). Growth area (pixels) corresponds to the surface occupied by the colonies in the spots. Each curve represents this surface evolution over the incubation period (up to 20 days) for a given cycloheximide concentration. Three representative profiles are illustrated: (**A**) low tolerance profile (strain CBS-74); (**B**) intermediate profile (strain L14195); and (**C**) resistant profile (strain L1752). All curves were obtained at low inoculum density (≈5–10 colonies/drop). Strains CBS-74, L14195, and L1752 belong to the Admixed D1/D2, A1, and D1 genetic groups, respectively.

Figure 3 shows the growth profiles extracted by the image-analysis pipeline (Area = f(time)) at different cycloheximide concentrations for three representative strains. Strain CBS-74 (Figure 3A) displayed a low tolerance profile, characterized by no detectable effect of low cycloheximide doses (0.005 and 0.01 g.L^−1^), for which growth was comparable to that observed in the control condition. At intermediate doses (0.05 and 0.1 g.L^−1^), growth initiation was delayed to day 8 (lag phase = 8 days), while at the highest doses (0.25 and 0.5 g.L^−1^), growth was completely inhibited over the 20-day monitoring period. Strain L14175 (Figure 3B) illustrated an intermediate profile: Although growth was visible at all concentrations, it became progressively slower and delayed as the cycloheximide concentration increased. Growth onset was delayed by 5 days at 0.05 g.L^−1^ and only occurred after 8 days at 0.5 g.L^−1^. Finally, strain L1752 (Figure 3C) exhibited a resistant profile, with growth that was similar yet sometimes delayed at the higher doses.

**Figure 4:**
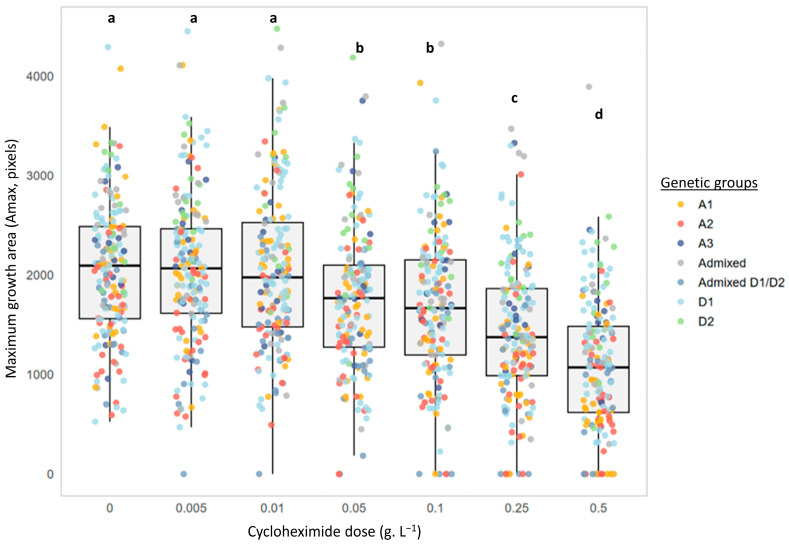
Effect of cycloheximide concentrations on the maximum growth area (Amax, pixels) reached by *B. bruxellensis*. Distribution of the growth area values of low inoculation density spots (≈5–10 colonies/drop) after 20 days of incubation of the 175 strains for each cycloheximide concentration (0 to 0.5 g.L^−1^) assayed. Each point represents the mean value (*n* = 3 assays) for a given strain and a given cycloheximide concentration. Colors indicate the genetic group to which each strain belongs (A1, A2, A3, Admixed, Admixed D1/D2, D1, and D2). The letters indicate significant differences (Kruskal–Wallis, *p*-value < 0.05).

In addition to the delay in colony appearance, Figure 3 indicates the maximum area reached by low-density deposits (Amax) after 20 days of incubation (J20). Once again, the profiles were different. Strains CBS-74 and L14195 showed a progressive reduction in Amax with increasing cycloheximide concentrations (Figure 3B), whereas the maximum area remained nearly unchanged across all tested concentrations for strain L1752 (Figure 3C). The impact of cycloheximide on the maximum area reached is, thus, strain-dependent. At the scale of the entire collection, Figure 4 confirms this trend. Regardless of the cycloheximide dose, Amax distributions remained highly heterogeneous. Nonetheless, the mean maximum area was clearly affected by cycloheximide. Across the 175 strains tested, Amax values were only slightly affected at low doses (0.005 and 0.01 g.L^−1^), with no significant differences compared to the control. By contrast, at intermediate doses (0.05 and 0.1 g.L^−1^), Amax values were significantly reduced, and at the highest doses (0.25 and 0.5 g.L^−1^), they decreased significantly (−30% to −50% for the mean value).

These results show that cycloheximide acts both by delaying colony detection (increase in lag phase) and by reducing spot expansion for certain strains (decrease in Amax), with variable effects depending on the strain. To illustrate this morphological impact more concretely, a comparison of colony appearance in the absence and presence of cycloheximide is provided in the supplemental data (Supplementary Materials Figure S2).

#### 3.1.3. Combined Effect of Cycloheximide Dose and Genetic Group on Growth Delay

**Figure 5:**
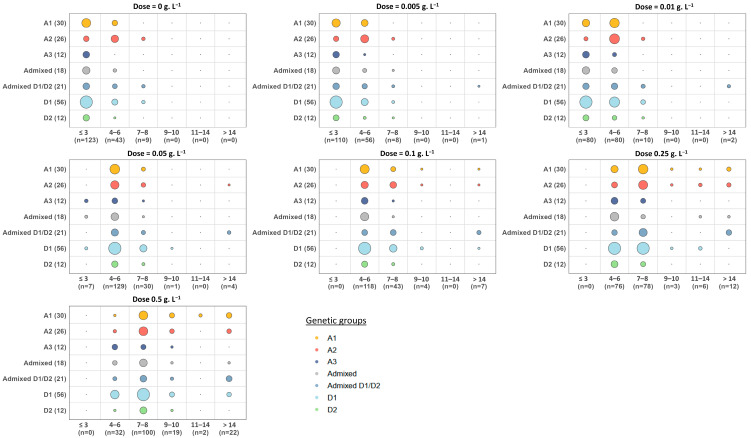
Distribution of the rough lag phases of 175 *B. bruxellensis* strains. The spots examined were those obtained at low inoculum density (≈5–10 colonies/drop). The 175 strains were grouped according to their genetic group (vertical axis, number of strains indicated in parentheses). Each panel corresponds to a different cycloheximide concentration (0 to 0.5 g.L^−1^). The *x*-axis indicates the lag phase classes (days), the bubble size being proportional to the number of strains concerned.

As previously mentioned, the analysis of rough lag phases on YPD solid medium highlights a strong variability among strains in the absence of cycloheximide. This phenomenon is further illustrated in Figure 5. Some strains displayed rapid growth, being detected after only three days of incubation on plates (≈70% of strains), and 95% were detectable after six days. However, a small fraction (≈4.6% of strains) only appeared after 7–8 days. With increasing doses of cycloheximide, the lag phases progressively shifted toward higher values (Figure 5). At low concentrations (0.005 and 0.1 g.L^−1^), most strains remained detectable on plates within a week, despite a slight delay when compared with growth without cycloheximide. Above 0.05 g.L^−1^, the delay for colony appearance became more pronounced. Indeed, only a few strains (*n* = 7) were detected before 3 days, while 74% appeared between 4 and 6 days, 17% between 6 and 8 days, and only a few after 8 days. At 0.1 g.L^−1^, the effect was even stronger, with nearly one strain out of three showing a lag phase longer than 6 days, meaning they would remain undetectable at day 6 if incubation was stopped. Finally, at the highest dose (0.5 g.L^−1^), about 13% of strains still failed to develop visible colonies after 14 days of incubation, and a similar proportion (≈12%) only became detectable between days 9 and 14.

**Figure 6:**
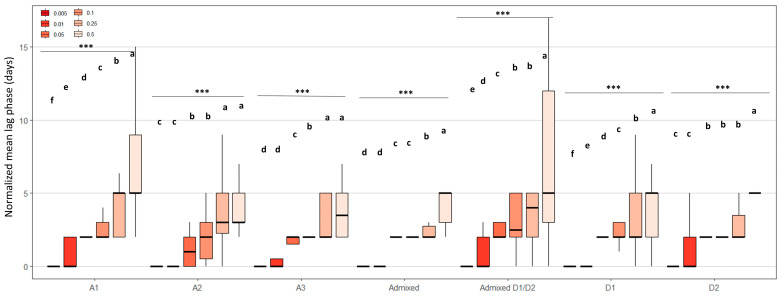
Combined effect of cycloheximide concentration on the normalized lag phases of *B. bruxellensis* grown on solid YPD medium. Results were obtained at low density (≈5–10 colonies/spot). Lag phases were normalized by subtracting, for each strain, the lag phase observed on the control plate without cycloheximide (0 g.L^−1^). The boxplot shows the distribution of normalized lag phases as a function of cycloheximide concentration (0.005 to 0.5 g.L^−1^) for each genetic group (A1, A2, A3, Admixed, Admixed D1/D2, D1, and D2). The letters indicate significant differences (Kruskal–Wallis, *p*-value < 0.05). *** indicates a statistically significant difference at *p* < 0.001.

To complete this analysis, we examined the lag phase after normalization in order to study the effect of cycloheximide on lag phase elongation independent of the heterogeneity of the basal lag phase on solid YP medium (Figure 6). Regardless of the genetic group, increasing cycloheximide concentration progressively extended the lag phases. For example, in group A1, lag phases increased from just a few days to up to more than 12 days for some strains at 0.5 g.L^−1^. A similar trend was observed in other groups, although with lower amplitude. Overall, the results illustrate a clear dose-dependent effect of cycloheximide on growth delay, with different intensities depending on the strain and genetic group. Despite strong intra-group heterogeneity, the most sensitive responses were generally observed in groups A1 and Admixed D1/D2, whose strains displayed the longest lag phases with increasing cycloheximide dose.

To identify the factors underlying the differences observed, a multifactorial parametric ANOVA including main effects and interactions was performed (Figure S3, Supplementary Materials). The results indicate that the variance in the normalized lag phase was mainly explained by cycloheximide dose (34%) and by the strain (21.2%). The interaction between dose and strain also contributed substantially (17.3%), highlighting that the effect of cycloheximide strongly depends on each isolate. Inoculum density (1.2%) and its interaction with the dose of cycloheximide (0.8%) contributed only marginally, while a large proportion of the variance remained unexplained (residuals, 25.5%). This residual fraction could reflect the influence of uncontrolled factors, such as the physiological state of the cells at the time of spotting on the plates.

### 3.2. Influence of Cycloheximide on the Detection of B. bruxellensis in Wine Samples

We next investigated whether these phenomena persisted, or could be amplified, under real oenological conditions, with the yeasts coming from samples in which they were exposed to additional stresses (acidity, ethanol content, or the presence of sulfites). For this section, the tests were performed on a subset of strains representative of the species’ genetic diversity.

#### 3.2.1. Characterization of Stress Levels in the Wine Samples

**Figure 7:**
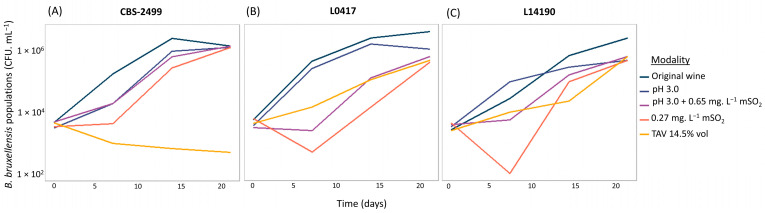
Growth of three *B. bruxellensis* strains (CBS-2499, L0417, and L14190) of cultivable counts over time (T0, T7, T14, and T21 days). (**A**) Strain CBS-2499, (**B**) strain L0417, and (**C**) strain L14190. Strains CBS-2499, L0417, and L14190 belong to the D1, A1, and Admixed D1/D2 genetic groups, respectively.

Firstly, in order to highlight the growth inhibition prevailing in the distinct wines, strain populations were determined on solid YPD medium in the absence of an antifungal agent after different incubation times in wine (T0, T7, T14, and T21 days). Cultivable populations are shown for three representative strains under the different oenological conditions (CFU. mL^−1^, Figure 7). The diploid strain CBS-2499 (Figure 7A) was able to grow in most modalities. However, its growth slowed down in all conditions when compared to the initial matrix (original wine) and was strongly impaired at high ethanol concentration (14.5% vol), in the presence of which populations progressively declined over time. In wine, added sulfites may combine with several compounds and become inactive. As a result, the biologically active doses of molecular SO_2_ (mSO_2_) should be considered. Strain L0417 (Figure 7B) was sensitive to both sulfite conditions (pH 3.0 + mSO_2_ 0.65 mg.L^−1^ and mSO_2_ 0.27 mg.L^−1^), as shown by a strong initial drop in populations within the first week after inoculation. Nevertheless, following an adaptation phase, growth resumed after seven days. Finally, strain L14190 (Figure 7C) maintained growth in most modalities, but its development remained more limited compared to the initial matrix. Unexpectedly, when strains resumed growth, no strong difference was observed between the two sulfite conditions.

#### 3.2.2. Combined Effect of Oenological Stress and Cycloheximide

To evaluate the combined effect of oenological constraints and cycloheximide on the extension of the lag phases independent of the inter-strain variability observed under control conditions, the lag phase was normalized by subtracting the lag phase observed for the same strain in the control condition (dose 0 g.L^−1^, original wine) for each strain x wine x cycloheximide dose assay. The cycloheximide doses used in this section were 0.05, 0.1, and 0.5 g.L^−1^.

**Figure 8:**
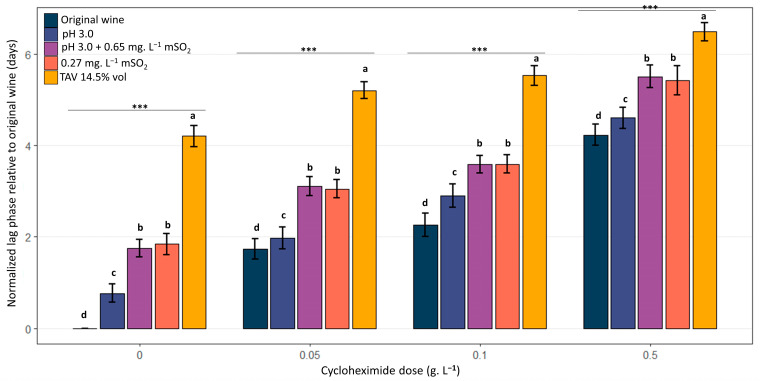
Combined effect of oenological stress in the sample and cycloheximide on the mean normalized lag phase observed for *B. bruxellensis* grown on solid YPD medium. Distribution of mean normalized lag phases values (*n* = 4 residence time ×10 strains, 3 replicates per assay) for each oenological modality, unmodified wine (original wine), acidified wine (pH 3.0), acidified wine supplemented with sulfites (resulting in 0.65 mg.L^−1^ mSO_2_), original wine supplemented with sulfites (resulting in 0.27 mg.L^−1^ mSO_2_), and original wine enriched to 14.5% vol alcohol as a function of cycloheximide concentration in the YPD solid medium (0, 0.05, 0.1, and 0.5 g.L^−1^). Lag phases (days) were normalized by subtracting, for each experiment (strain, modality, residence time in wine, and cycloheximide concentration in the plating medium), the lag phase value observed for the same strain in the control condition (original wine, sampled at the corresponding residence time in wine and plated in the absence of cycloheximide). The letters indicate significant differences (Kruskal–Wallis, *p*-value < 0.05). *** indicates a statistically significant difference at *p* < 0.001.

Figure 8 compares the impact of the different oenological conditions on the growth delay observed for a given cycloheximide concentration. The modalities were not equivalent: conditions including SO_2_ (pH 3.0 + mSO_2_ 0.65 mg.L^−1^ and mSO_2_ 0.27 mg.L^−1^) and, most notably, the high ethanol condition (14.5% vol) induced significantly longer lag phases compared to the initial matrix or simple acidification (pH 3.0). Concretely, when strains were exposed to 0.5 g.L^−1^ cycloheximide, colony detection required an additional 3–5 days of incubation for wine samples containing 14.5% vol ethanol and 2–3 extra days for those harboring sulfites, in comparison with those made of the unmodified wine matrix. Thus, certain oenological stresses amplify the inhibitory effect of cycloheximide by increasing the lag phase. The hierarchy of modalities (in terms of stress intensity) remained consistent regardless of the cycloheximide dose: 14.5%vol ethanol > SO_2_ > pH 3.0 > original wine. The results show a cumulative effect of cycloheximide and oenological stresses on the time required for growth resumption on plates after spreading. The trend was dose-dependent.

**Figure 9:**
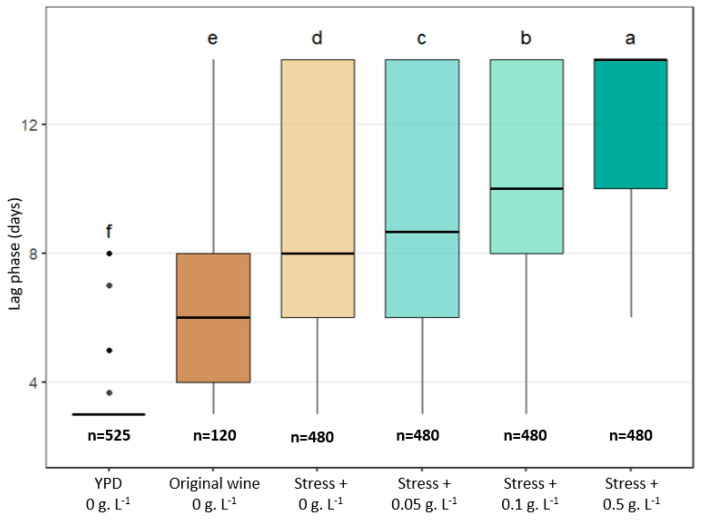
Global distribution of raw lag phases (days) obtained under the different experimental conditions examined in this paper. For YPD medium, each point represents the lag phase measured after transfer of YPD culture samples to YPD plates containing 0 g.L^−1^ of cycloheximide (175 strains × 3 *inoculum density* = 525). For wine conditions, included points correspond to the lag phases obtained with the 10 strains × 4 residence time × 3 inoculum densities (=120), and the stress assays gather the lag phases observed for the 4 modified wines, sampled at 4 residence times and spotted at 3 inoculum densities (480). The values “*n* = …” indicate the total number of assays taken into account for representation. The letters indicate significant differences (Kruskal–Wallis, *p*-value < 0.05).

To sum up, Figure 9 illustrates the evolution of the raw lag phase distribution from the most efficient growth recovery conditions evaluated in this paper to the most difficult one. The shortest lag phases were observed when cells were transferred directly from liquid YPD to solid YPD medium without antibiotic: in this case, the vast majority of strains (166/175) formed visible colonies within 6 days, and 123/175 formed colonies within 3 days. When cells originated from relatively permissive wine samples (the original wine used in this study), that is, wines, in which *B. bruxellensis* can grow rapidly and maintain good physiological fitness [16], the lag phases were significantly longer (mean of 6 days, with high inter-strain variability), reflecting the physiological adjustment required to switch from the wine environment to the plate nutrient-rich YPD medium. When samples were taken from wines subjected to additional stresses (acidity, ethanol, and sulfites), the delay in colony appearance on the YPD plates increased further (mean of 8 days, with maximal dispersion), even in the absence of antibiotic in the solid medium. The presence of cycloheximide further accentuated this growth delay in a dose-dependent manner and with variable magnitude depending on the strain, resulting in mean lag phases of approximately 10 days at 0.1 g.L^−1^ and 14 days at 0.5 g.L^−1^.

## 4. Discussion

The major result of this work is the strong intraspecies diversity observed in the tolerance of *B. bruxellensis* to cycloheximide. Indeed, all the strains can be considered tolerant, as all grow at low antibiotic concentration, contrarily to *Saccharomyces cerevisiae*, which is always inhibited [32,36]. However, some strains appear to be much more tolerant than others. If growth is observed for all strains on solid media containing cycloheximide, it can sometimes be less efficient and delayed. These effects of cycloheximide are dose-dependent and visible for all seven genetic groups studied. However, the magnitude of the response greatly varies among strains.

As a side part of our study, we first observed a great heterogeneity in the growth on YPD solid medium without cycloheximide between the 175 strains. When they originate from liquid YPD medium samples, some strains form detectable colonies in less than three days, while others show slower growth and appear after six to eight days of incubation, notably including those in the group of wine strains A2 (formerly “Wine1” or “AWRI1499-like”). This phenomenon may be linked to higher recovery times needed for shifting from planktonic to biofilm life or to distinct nutritional requirements or distinct adaptation to the incubation temperature assayed.

When cycloheximide is added to YPD medium, some strains can still be detected quickly (colonies visible in less than a week), even at the highest antibiotic concentrations (0.25 and 0.5 g.L^−1^), reflecting high tolerance. Others show a gradual lengthening of their latency phase as the concentration increases but without inhibition of the final expansion of the spots. Finally, a third group of strains comprises even less tolerant strains, completely inhibited at the highest doses and sometimes at intermediate doses (0.05 and 0.01 g.L^−1^). The two genetic groups A2 and D1, comprising most of the Bordeaux red wine strains [40], appear to be among the most tolerant, in the sense that their latency phase seems less extended by the antibiotic than for other groups. Nevertheless, a strong intra-group heterogeneity remains, and tolerance to cycloheximide seems less clearly associated with the genetic group than tolerance to sulfites or other stresses encountered in wine. Indeed, many studies have highlighted that *B. bruxellensis* strains differ greatly in their tolerance to stress factors [11,13,14,27,41] and that part of this phenotypic diversity is associated with genetic groups [6,9,10,14,42,43], the strains of group A2 being generally more resistant to abiotic wine stresses than the strains of group D1 mentioned above.

Our results confirm previous observations obtained by other authors with a lower number of strains and lower concentration ranges (0.01 to 0.1 g.L^−1^, refs. [30,32,37]). By expanding the cycloheximide tolerance analysis to doses up to 0.5 g.L^−1^ and using a panel of 175 *B. bruxellensis* strains, our study demonstrates that the variability in sensitivity of the species is even more marked than previously estimated and even more visible at high doses. Beyond the growth onset time, cycloheximide also influences colony final size. Ref. [32] had already shown that even for strains able to quickly grow in the presence of cycloheximide, the increase in dose led to smaller colonies after seven days of incubation, a difference that can be lowered if incubation is prolonged. Our results confirm this observation but reveal that, for some strains, the reduction in the maximum area (reflecting the size of the colonies) persists even after 20 days of incubation and is accentuated with the cycloheximide dose increase.

The cell density on the plate also influences the apparent tolerance to cycloheximide: basically, the higher the density, the faster the colonies’ appearance and the shorter the latency phase induced by cycloheximide. Cell density is known to modulate the response of microorganisms to various types of stresses and antimicrobial agents and to modulate adaptive behaviors such as biofilm formation. Ref. [44] showed that higher *S. cerevisiae* densities reduced the yeasts’ adaptation time and increased their tolerance to weak acid stress (acetic, formic). Ref. [45] observed that the increase in *Candida albicans* cell density increased the minimum inhibitory concentrations (MICs). Ref. [46] showed that increasing *Pseudomonas aeruginosa* inoculum concentration favored the formation of biofilms denser and more resistant to antibiotics. Cooperative phenomena promoting SO_2_ tolerance have also been suggested in planktonic *B. bruxellensis* cultures [28]. Similar phenomena could explain the shorter latency phases obtained at high densities in our study.

If, on the one hand, cooperation may accelerate the adaptation to cycloheximide after plating, on the other hand, the occurrence of stress factors in the spotted samples delays the growth start. The oenological samples examined in this paper and displaying stress situations (acidity, presence of sulfites, or ethanol level) showed an amplified inhibitory effect of cycloheximide. The extent of growth delay varies according to the cycloheximide dose and the oenological constraint. In extreme cases, at high antibiotic doses, some strains become visible only after 8 to 14 days, while others are detectable in less than one week. Otherwise stated, in the presence of 0.5 g.L^−1^ of cycloheximide, the lag phase is extended from three to five days when the studied samples come from wines with high alcohol content and from two to three days if the analyzed wines contain sulfites. Anticipating these phenomena, ref. [47] modulated the cycloheximide dose to isolate *B. bruxellensis* from wine samples collected in several domains. The antibiotic dose used was 0.1 g.L^−1^ for old vintages (<1990) to avoid damage of the yeasts stored in the wines for long, and 0.5 g.L^−1^ was used to isolate *B. bruxellensis* in more recent vintages. Anticipating the risk of false negatives linked to growth inhibition, she prolonged the plates’ incubation for up to 3 weeks. A few years earlier, refs. [48,49] recommended the use of a selective medium containing 0.5 g.L^−1^ cycloheximide and an incubation of at least 10 days to measure *B. bruxellensis* populations in young red wines.

The present paper goes further in calling for caution in setting up routine detection methods for *B. bruxellensis* in wine. In laboratories, the selective media used for *B. bruxellensis* detection usually contain between 0.01 and 0.05 g.L^−1^, but some analyses are performed with higher concentrations (≥0.1 g.L^−1^) to increase selectivity. In practical terms, the optimal cycloheximide concentration should be adapted to the winemaking stage. Low doses (≈0.01 at 0.05 g.L^−1^) are generally sufficient for wines in aging or storage phases, where yeasts are not so numerous in the wine. However, at these stages, wine stresses may be very high. By contrast, a cycloheximide dose of 0.5 g.L^−1^ seems particularly suitable for wine analyses conducted in pre-fermentation and fermentation phases, where populations of yeast other than *B. bruxellensis* are present and selectivity is required. Whatever the winemaking stage considered, prematurely stopping incubation (e.g., at 6 days, as frequently done routinely in analytical laboratories) carries a high risk of false negatives or a high risk of underestimation of the populations present, especially when the wine is under stress and when the population in the sample is low (due to the density effect mentioned above). We, thus, recommend letting the plates incubate for between 6 and 10 days or even more, with regular monitoring, in case of low colony development.

## 5. Conclusions

This paper highlights the precautions needed regarding the incubation period when using solid selective media containing cycloheximide for the detection and quantification of *B. bruxellensis* populations. More precisely, when the use of high doses (≥0.1 g.L^−1^) of cycloheximide seems relevant to limit the growth of other non-Saccharomyces yeasts, or when the wines examined display high alcohol or sulfite doses, incubation times should be extended (10 days or even up to 3 weeks) in order to limit false negatives.

## Data Availability

The raw data supporting the conclusions of this article will be made available by the authors on request.

## Supplementary Materials

The following supporting information can be downloaded at: https://www.mdpi.com/article/10.3390/microorganisms13112597/s1, Table S1: List of the yeast strains in this work; Figure S1: Normalized lag phases (days) for 175 strains grouped by inoculation density and cycloheximide dose (0.005 to 0.5 g.L^−1^); Figure S2: Effect of cycloheximide on the morphological appearance of colonies; Figure S3: Contribution of the different factors to the total variance of normalized lag phases in *B. bruxellensis*; Figure S4: Combined effect of oenological stress in the sample and cycloheximide on the mean normalized lag phase observed for *B. bruxellensis* grown on solid YPD medium.

## Abbreviations

The following abbreviations are used in this manuscript:

YPD	yeast peptone dextrose
CFU	colony-forming unit
