# Supplementary material for: Brettanomyces bruxellensis Strains Display Variable Resistance to Cycloheximide: Consequences on the Monitoring of Wine
Source: Microorganisms. 2025 Nov 14;13(11):2597. doi: 10.3390/microorganisms13112597 (PMC12654844; doi:10.3390/microorganisms13112597)
Supplement: Supplementary file 1 [file microorganisms-13-02597-s001.zip › Figure S1.pdf]

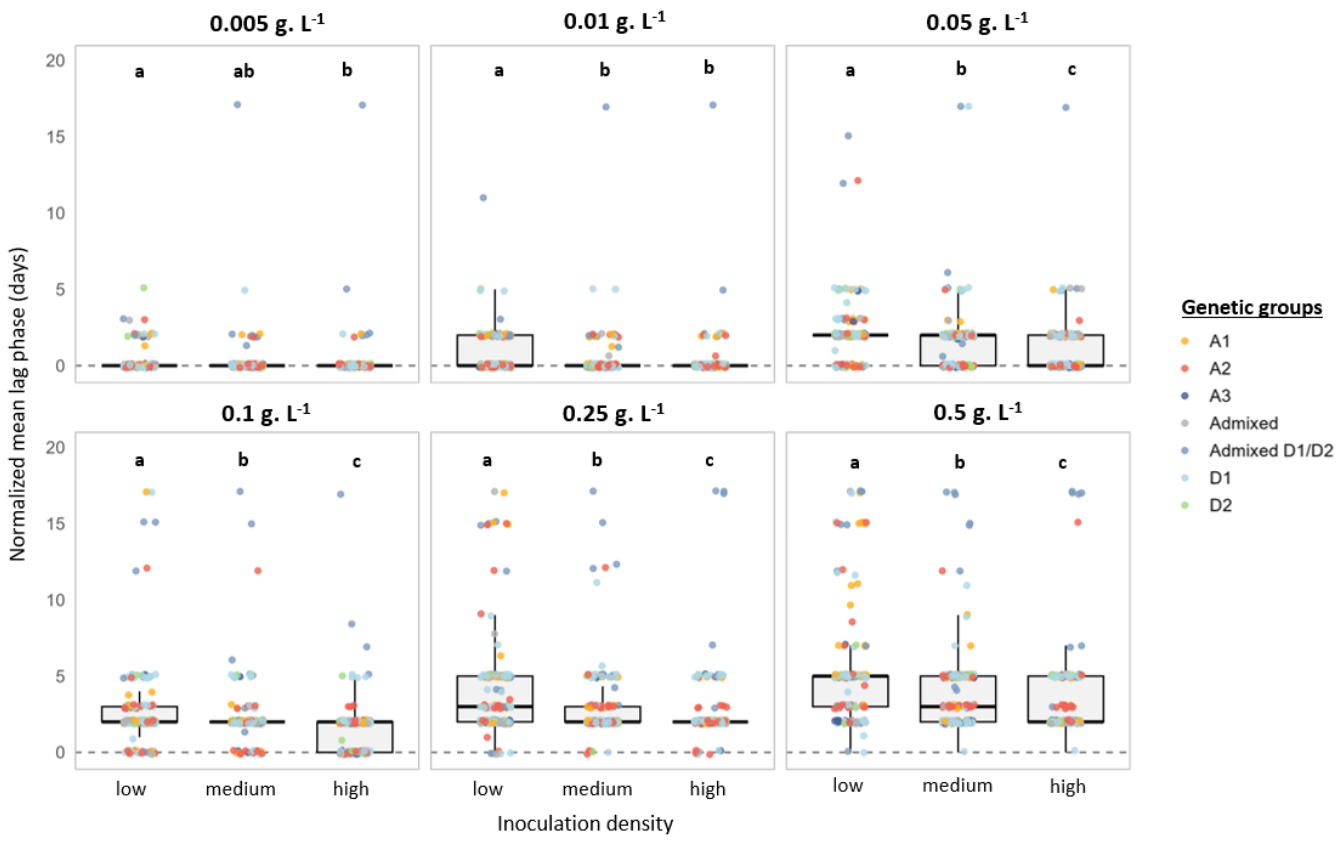

Figure S1. Normalized lag phases (days) for 175 strains grouped by inoculation density and cycloheximide dose (0.005 à 0.5 g.L^{-1}). Lag phases were normalized by subtracting, for a given strain and a given density, the lag phase observed on the corresponding spot in the control plate without cycloheximide (0 g.L^{-1}). Each boxplot represents the distribution of mean normalized lag values (n=3 for each dot) for the 175 strains, 6 cycloheximide concentrations and three inoculation densities: low (≈ 5-10 colonies/drop), medium (≈ 50-100 colonies/drop) and high (≈ 500-1000 colonies/drop). Dots are coloured according to the genetic group of corresponding strain (A1, A2, A3, Admixed, Admixed D1/D2, D1 et D2). The letters indicate significant differences (Kruskal Wallis, p-value < 0.05).
