# Supplementary material for: Brettanomyces bruxellensis Strains Display Variable Resistance to Cycloheximide: Consequences on the Monitoring of Wine
Source: Microorganisms. 2025 Nov 14;13(11):2597. doi: 10.3390/microorganisms13112597 (PMC12654844; doi:10.3390/microorganisms13112597)
Supplement: Supplementary file 1 [file microorganisms-13-02597-s001.zip › Figure S2.pdf]

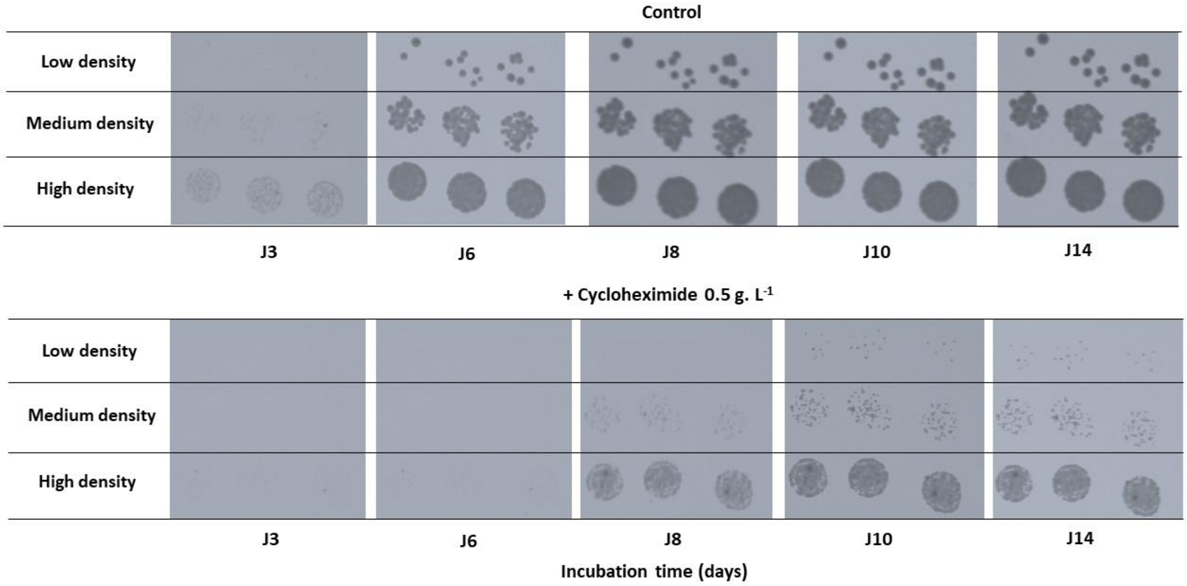

Figure S2. Effect of cycloheximide on the morphological appearance of colonies. Comparison of the growth of a strain (L0516) cultivated in the absence (control) or presence of 0.5 $g.L^{-1}$ cycloheximide, at three inoculation densities: low (≈ 5-10 colonies/drop), medium (≈ 50-100 colonies/drop) and high (≈ 500-1000 colonies/drop). The appearane of the colonies was monitored at different incubation times (J3, J6, J8, J10 and J14).
