# Supplementary material for: Brettanomyces bruxellensis Strains Display Variable Resistance to Cycloheximide: Consequences on the Monitoring of Wine
Source: Microorganisms. 2025 Nov 14;13(11):2597. doi: 10.3390/microorganisms13112597 (PMC12654844; doi:10.3390/microorganisms13112597)
Supplement: Supplementary file 1 [file microorganisms-13-02597-s001.zip › Figure S3.pdf]

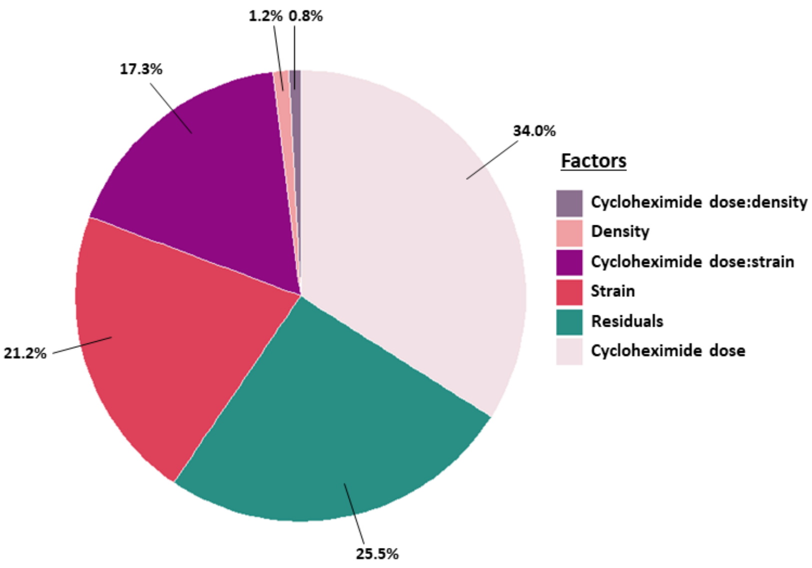

Figure S3. Contribution of the different factors to the total variance of normalized lag phases in *B. bruxellensis*. The percentages indicate the proportion of variance explained by each factor, calculated from a parametric multifactorial analysis performed on the full dataset of normalized lag phases obtained from liquid YPD cultures.
