# Supplementary material for: Brettanomyces bruxellensis Strains Display Variable Resistance to Cycloheximide: Consequences on the Monitoring of Wine
Source: Microorganisms. 2025 Nov 14;13(11):2597. doi: 10.3390/microorganisms13112597 (PMC12654844; doi:10.3390/microorganisms13112597)
Supplement: Supplementary file 1 [file microorganisms-13-02597-s001.zip › Figure S4.pdf]

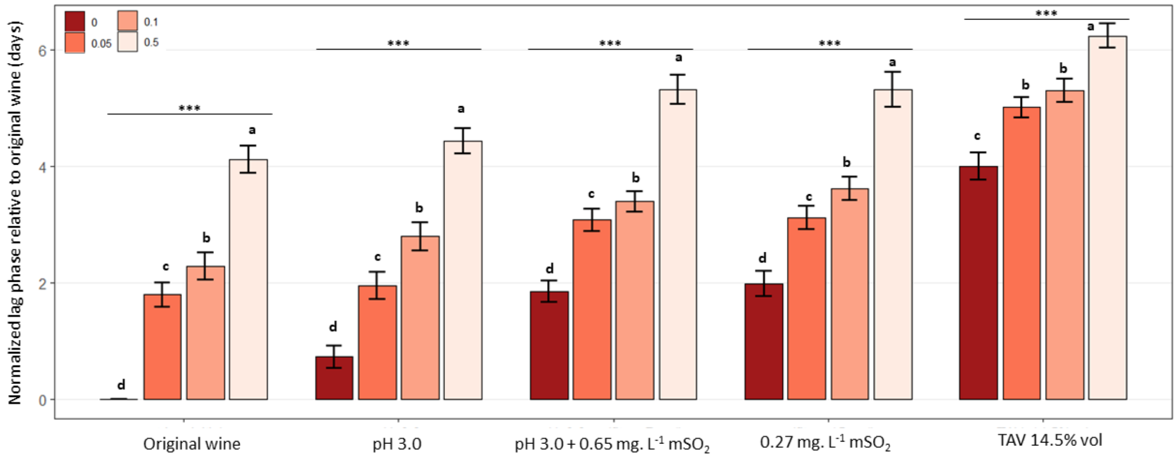

Figure S4. Combined effect of oenological stress in the sample and cycloheximide on the mean normalized lag phase observed for *B. bruxellensis* grown on solid YPD medium. Distribution of mean normalized lag phases values (n=4 residence time x10 strains, 3 replicates per assay) as a function of cycloheximide concentration, for each wine sample assayed: unmodified wine (original wine), acidified wine (pH 3.0), acidified wine supplemented with 7 mg.L^{-1} free SO_2, original wine supplemented with 15 mg.L^{-1} free SO_2 and original wine enriched to 14.5% vol alcohol. Lag phases (days) were normalized by subtracting, for each experiment (strain, modality, residence time in wine, and cycloheximide concentration in the plating medium), the lag phase value observed for the same strain in the control condition (original wine, sampled at the corresponding residence time in wine and plated in the absence of cycloheximide). The letters indicate significant differences (Kruskal Wallis, p-value <0.05).
