# Supplementary material for: Brettanomyces bruxellensis Strains Display Variable Resistance to Cycloheximide: Consequences on the Monitoring of Wine
Source: Microorganisms. 2025 Nov 14;13(11):2597. doi: 10.3390/microorganisms13112597 (PMC12654844; doi:10.3390/microorganisms13112597)
Supplement: Supplementary file 1 [file microorganisms-13-02597-s001.zip › Table S1.pdf]

Table S1. List of the yeast strains in this work.

Strains (*B. bruxellensis*)
**A1 (30):** 2OT13-05; 2OT13-07; 2OT14-01; C9_D1_115; C5_A3_ 28; GB62; ISA2397; CRBO L0417; CRBO L0422; CRBO L0516; CRBO L14173; CRBO L14183; CRBO L14192; CRBO L14195; CRBO L17105*; CRBO L17106; CRBO L17112; CRBO L17117; CRBO L17118; CRBO L17119*; CRBO L17120; CRBO L1748, CRBO L1750; CRBO L1771; LB15110G; NL058; WLP645; YJS5397; YJS5400; YJS5454
**A2 (26):** 13-Mx-nC3d1; 33_2; CRBO L0424*; CRBO L14156; CRBO L14174; CRBO L14175; CRBO L17109; CRBO L17113; CRBO L1713; CRBO L1728; CRBO L1746; CRBO L1792*; CRBO L1793*; CRBO L1795*; NCAIM Y 00666; UCD_VEN_ 3993; VP1541; YJS5408; YJS5434; YJS5445; YJS5459; YJS5469; YJS5473; YJS5476; YJS5478; YJS5487
**A3 (12):** C-00346; C_00348; CBS 8027; CBS 6055; CRBO L17103*; CRBO L17104*; CRBO L17108; CRBO L1718; MUCL 27706; NCYC-3426; NCYC 3441; UWOPS_92_298_4
**Admixed (18):** OENO_15_1; Ch1; DBVPG 7280; ISA1601; CRBO L14165; CRBO L14169; CRBO L1778; VP1547**; YJS5301; YJS5334; YJS5349; YJS5384; YJS5398; YJS5402; YJS5406; YJS5413; YJS5417; YJS5431
**Admixed D1/D2 (21):** OENO_33_1; CBS-78; CBS 74; CBS3025; Gamay 329 CM5; GB17; ISA2211; CRBO L0308; CRBO L14190; CRBO L1710; CRBO L17107*; CRBO L1731; CRBO L1733; CRBO L1739; CRBO L1783; NCYC 2818; NCYC 3050; NL064; VP1544; Y1413; YJS5382
**D1 (56):** 12_LT_VGC3_c10; 1961_MX_M1_E2; 2OT13-02; CBS 5512; CBS 2499; CDR12; DBVPG 7279; CRBO L0420; CRBO L0469; CRBO L0611; CRBO L14160; CRBO L14163; CRBO L14168; CRBO L14186; CRBO L1703; CRBO L17100; CRBO L1711; CRBO L17110; CRBO L17116; CRBO L1715; CRBO L1719; CRBO L1720; CRBO L1722; CRBO L1732; CRBO L1737; CRBO L1745; CRBO L1751; CRBO L1752; CRBO L1772; CRBO L1791; CRBO L1794; PYCC 4801; SJ12-4; UCD_VEN_2980; UCD_VEN_2987; C_05 794; C_16959; YJS5302; YJS5319; YJS5320; YJS5345; YJS5347; YJS5357; YJS5373; YJS5385, YJS5392; YJS5422; YJS5426; YJS5440; YJS5447; YJS5449; YJS5453; YJS5458; YJS5461; YJS5479; YJS5485
**D2 (12):** CRBO L0463; CRBO L0467; CRBO L14181; CRBO L1738; PYCC 5249; YJS5310; YJS5340; YJS5344; YJS5363; YJS5368; YJS5407; YJS5420

* Strains not sequenced by [10]: group attribution was based on microsatellite analysis according to [6]
